# Molecular Chimera in Cancer Drug Discovery: Beyond Antibody Therapy, Designing Grafted Stable Peptides Targeting Cancer

**DOI:** 10.1007/s10989-025-10690-6

**Published:** 2025-02-17

**Authors:** Arpan Chowdhury, Prajesh Shrestha, Seetharama D. Jois

**Affiliations:** Department of Pathobiological Sciences, School of Veterinary Medicine, Louisiana State University Baton Rouge, Skip Bertman Drive, Baton Rouge, LA-70803 USA

**Keywords:** Targeted therapy, Cancer, Small molecules, Cyclotides, Sunflower trypsin inhibitor

## Abstract

**Background:**

Several cancer therapies are being developed, and given the variability of different cancer types, the goal of these therapies is to remove the invasive tumor from the body, kill the cancer cells, or else retard the growth. These include chemotherapeutic agents and targeted therapy using small molecules and antibodies. However, antibodies can generate an immune response upon repeated administration, and producing antibodies could be expensive.

**Purpose:**

The purpose of this review is to describe different therapeutic approaches utilized for cancer therapy, the current therapeutic approaches, and their limitations. As a novel strategy to combat cancer, designing new stable peptide scaffolds such as cyclotides and sunflower trypsin inhibitors (SFTI) is described.

**Results:**

Stable peptides that can target proteins can be used as therapeutic agents. Here, we review the utilization and amalgamation of plant-based peptides with biological epitopes in designing molecules called “Molecular Chimeras” using a grafted peptide strategy. These cyclic peptides can bind to target receptors or modulate protein-protein interactions as they bind with high affinity and selectivity. Grafted peptides also possess better serum stability owing to the head-to-tail cyclization and other structural modifications.

**Conclusion:**

Stable cyclic peptides outweigh the other biologicals in terms of stability and manufacturing process. Peptides and peptidomimetics can be used as therapeutic agents, and these molecules provide alternatives for biologicals and small molecule inhibitors as drugs.

## Introduction

Based on the projections provided by the American Cancer Society for the year 2024, it is anticipated that there will be an estimated two million incidences of newly diagnosed cancer cases and more than 600,000 fatalities resulting from cancer-related causes within the United States (Siegel et al. 2024). Though surgery is the most effective treatment in the early stages of cancer progression, it has limitations in the later stages; similarly, radiation therapy can damage healthy cells, organs, and tissue (Padma 2015; Debela et al. 2021). Several novel and new therapeutic approaches are proposed that are still in preclinical settings, and some of them are in clinical trials. Traditionally chemotherapeutic agents and their modifications using new delivery strategies, and antibody-targeted therapies have succeeded but face some challenges. Possible new approaches beyond antibody therapy are proposed. In this review, we will discuss different therapeutic approaches utilized for cancer therapy, the current therapeutic approaches, and their limitations. We will then focus on peptides and peptidomimetics as a novel strategy to combat cancer with a primary focus on stable peptide scaffolds such as cyclotides and sunflower trypsin inhibitors (SFTI) as a molecular scaffold in the development of cancer therapies. Lastly, we will describe the advantages and challenges of developing these novel therapies.

## Limitations of Chemotherapeutic Agents

Most of these chemotherapeutic agents are synthetic small molecules or molecules derived from natural products and affect rapidly growing cells, including cancer and non-cancerous cells like cells in the gastrointestinal (G.I.) tract, bone marrow, and hair follicles. Consequently, there are widespread toxicities, including myelosuppression, nausea, vomiting, diarrhea, alopecia, etc. (Muhammad T. Amjad, 2023). Furthermore, the bioavailability of most chemotherapeutic agents is compromised due to their poor water solubility which can be overcome by solubility enhancers like Cremophor. For instance, polyoxyethylated castor oil or Cremophor EL in absolute ethanol is used in Paclitaxel formulation to increase its aqueous solubility for ease in intravenous delivery (Ma et al. 2021).

### Targeted Therapy

Targeted therapy is the cornerstone of precision medicine, which exploits medication or chemicals that target proteins or genes that affect cancer’s growth, division, and dissemination (Padma 2015). Monoclonal antibodies (mAbs) and small molecule medication are primarily used and occupy most of the targeted therapy market. Nevertheless, developing personalized therapeutic approaches to fight cancer is paramount due to individual variability. According to the Targeted Therapy Global Market Report 2024, the need for targeted therapy is anticipated to increase at a compound annual growth rate (CAGR) of 7.5%, from $97.72 billion in 2023 to $105.06 billion in 2024. By the year 2028 this stat is expected to reach $140.77 billion at a compound annual growth rate (CAGR) of 7.6% indicating that as the prevalence of cancer increases, these numbers are bound to ascend (*Targeted Therapy Market SIze*, *Trends*, *Growth And Analysis Report 2033*).

Some significant examples of various categories of targeted therapy against several cancer types are signal transduction inhibitors, hormone-based treatments, gene expression regulators, apoptosis inducers, angiogenesis inhibitors, immunotherapies, and toxin delivery agents (Wabel and Maitham 2019). Furthermore, various passive or active targeted delivery systems have been developed to improve the selective distribution of chemotherapeutic agents, increase their cytotoxicity against cancer cells, and decrease the systemic toxicity profile. While passive targeting uses encapsulated delivery methods to benefit from the tumor microenvironment’s improved penetration and retention effect, active targeting focuses on receptor-mediated interactions such as cell surface ligands coupled to the therapeutic moiety (Wabel and Maitham 2019). Additionally, passive targeting utilizes delivery methods to enhance the pharmacokinetic properties of anticancer medicines by taking advantage of the tumor microenvironment’s features, such as encapsulating chemotherapeutic medications in a nanocarrier, allowing for increased drug levels in tumors to induce cytotoxicity against cancer cells while reducing adverse effects (Stolarz et al. 2022). Another example of passive targeting is albumin-bound Paclitaxel. Paclitaxel, despite being formulated in Cremophor EL and dehydrated ethanol (50:50, v/v) to overcome its limited aqueous solubility, still has some serious adverse effects associated with Cremophor EL and ethanol (Ma and Mumper 2013). Hence, albumin-bound paclitaxel (Nab-Paclitaxel) was developed, providing better pharmacokinetic and pharmacodynamic characteristics. The Food and Drug Administration (FDA) authorized nab-paclitaxel for metastatic breast cancer in 2005 based on a phase III clinical trial that compared Paclitaxel to nab-paclitaxel. It was found that nab-paclitaxel (Abraxane) was more effective than Paclitaxel in metastatic breast cancer treatment (Gradishar et al. 2005).

### Small Molecule Inhibitors

Small molecule inhibitors are molecules less than 500 Da that restrict the function of target proteins by precisely binding to the binding pocket of ligands competitively (Liu et al. 2022). Because of their size and lipophilicity, they may easily cross the plasma membrane of the cells and are accessible orally (Debela et al. 2021). These molecules have a wide range of targets, such as kinases, enzymes, and proteasomes. Protein kinases are the most commonly studied small molecule inhibitors as they are crucial in cell division, growth, and proliferation (Zhong et al. 2021). As of 2022, over 72 FDA-approved medicinal agents target over a dozen distinct protein kinases, out of which 40 of them target tyrosine kinases, 12 inhibit protein-serine/threonine kinases, four target dual specificity protein kinases (MEK1/2), and 16 inhibit nonreceptor protein-tyrosine kinases (Roskoski 2023). Similarly, 62 molecules are anti-neoplasm (50 against solid tumors, eight against nonsolid tumors, and four against solid and nonsolid tumors). Similarly, as per the Protein Kinase Inhibitor Database (PKIDB) (Carles et al. 2018; Bournez et al. 2020), as of March 2024, there are 372 small molecule protein kinase inhibitors in clinical trials.

Furthermore, epidermal growth factor receptor (EGFR) inhibitor is another major class of small molecule tyrosine kinase inhibitor, given that approximately 15% of lung cancer cases in the U.S. are EGFR-positive, belonging to the NSCLC adenocarcinoma subtype (Roskoski 2023), Particularly, EGFRs have four members: human epidermal growth factor receptor (HER1/EGFR), HER2, HER3, and HER4. These isoforms share structural and sequence similarities, containing extracellular, transmembrane, and intracellular tyrosine kinase domains. Upon activating these receptors, they form homo and heterodimers to induce autophosphorylation of the intracellular tyrosine kinase domain (Hsu and Hung 2016). While HER2 lacks a ligand binding site, HER3 is known to lack tyrosine kinase activity and relies on other receptors for its activity. These phosphorylated residues function as docking sites for a variety of adapter and scaffolding proteins, inducing a wide range of downstream signaling pathways, including PI3K/AKT, Ras/MEK/ERK, PLC/PKC, and JAK/STAT, which contribute to the overall survival of the tumor. Alongside activating these pathways, it also upregulates the expression of genes that activate epithelial to mesenchymal transition responsible for cancer migration and invasion, leading to metastasis. The particular ligand, dimeric complexes, and proteins linked to the tyrosine phosphorylated residues in the C-terminal tail of the receptors control the strength and consequence of activated signaling cascades (Zhong et al. 2021). In-frame deletions in exon 19 and the L858R point mutation in exon 21 are the two most frequent activating mutations (Tomasello et al. 2018).

Several generations of Tyrosine kinase inhibitors (TKIs) target the EGFR. Gefitinib, erlotinib, and icotinib are some of the first-generation drugs for treating NSCLC patients with EGFR-activating mutations (Exon 19 deletion and exon 21 L858R) (Tomasello et al. 2018). Similarly, Afatinib and dacomitinib are irreversible second-generation inhibitors intended to treat the EGFR T790M mutation. Osimertinib and almonertinib are examples of third-generation EGFR TKIs that precisely target the T790M mutation and EGFR-activating mutations while avoiding the wild-type EGFR, unlike the second-generation inhibitors, which played a crucial role in overcoming the side effect of second-generation TKIs. Dual-target inhibitors, e.g., lapatinib and neratinib, block both EGFR and HER2 and are used in breast cancer treatment. A recently licensed HER2 inhibitor, tucatinib, treats advanced or metastatic HER2-positive breast cancer (Zhong et al. 2021).

Nevertheless, resistance is the major issue in developing small molecular TKI treatments. After receiving treatment for 9 to 14 months, acquired resistance to EGFR TKIs appears. Numerous genetic anomalies have been proposed as potential factors for EGFR TKI acquired resistance, such as secondary EGFR mutations, activation of parallel signaling pathways, histological changes, and activation of downstream signaling pathways. Research shows that exon 20 mutations confer most EGFR TKIs resistance, whereas exon 18 mutations are uncommon and primarily activating (Tomasello et al. 2018). EGFR C797S mutation is shown to mediate resistance against third-generation EGFR inhibitors. Similarly, resistant samples showed both *cis* and *trans* alteration of C797S and T790M. In the case of transmutation, the combination of first and third-generation EGFR TKIs is a successful therapy option. On the other hand, cis mutations are resistant to all approved EGFR TKIs, which have been a focus for the development of fourth-generation TKIs (Zhong et al. 2021).

### Antibodies

Immunotherapy can be used to treat cancer in a variety of ways (Havel et al. 2019). It can activate or enhance the immune system’s natural defenses to locate and fight cancer cells or create compounds in the lab resembling immune system components to restore and increase the immune system’s ability to find and attack cancer. Our immune system maintains track of every substance regularly found in the body and attacks any new ones it does not recognize. This property of the immune system helps protect our body against infection and cancer. Checkpoint inhibitors, Chimeric antigen (CAR) T cell treatment, Cytokines, Immunomodulators, cancer vaccines, Monoclonal antibodies, and Oncolytic viruses are some of the immunotherapies being researched (Riley et al. 2019). In this section, we will be discussing further about monoclonal antibodies.

Antibodies can target the cancer cell by interacting with surface antigens differentially expressed in cancer and obstruct the growth and survival pathways of ligand receptors responsible for their survival. In addition, complement-mediated cytotoxicity, antibody-dependent cellular phagocytosis, and antibody-dependent cellular cytotoxicity also play a crucial role in eliminating tumor cells (Weiner et al. 2012). The monoclonal antibodies that can work by themselves are naked monoclonal antibodies; however, antibodies can also be used as homing molecules to deliver chemotherapeutic drugs or radioactive material to target the cancer cell, known as conjugated antibodies. They can attach to the antigen on cancer, non-cancerous, or even free-floating proteins(Choi et al. 2024).

To reiterate, antibodies can enhance the body’s immune response against cancer by binding to cancer cells and serving as a signaling mechanism. For instance, alemtuzumab, a humanized, IgG1 kappa monoclonal antibody, recognizes the cell surface glycoprotein CD52 expressed on normal and malignant cells and is used in the therapeutic management of chronic lymphocytic leukemia (CLL) (Demko et al. 2008; Robak 2008). The CAM307 study demonstrates the superiority of alemtuzumab over chlorambucil in terms of overall response in patients with B-cell lymphocytic leukemia (83% vs. 55%) and complete remission rate (24% vs. 2%). These findings led to the approval of alemtuzumab by the FDA for treating B-cell lymphocytic leukemia in September 2007. Apart from that some monoclonal antibodies boost the immune response by targeting the immune checkpoint. e.g., pembrolizumab, a humanized IgG4 kappa monoclonal antibody, suppresses the programmed death-1 (PD-1) receptor which is a key regulator of immunological checkpoints in the tumor microenvironment (Dang et al. 2016). In 2014, the Food and Drug Administration (FDA) authorized pembrolizumab to treat individuals with unresectable or metastatic melanoma. In the same year, it was designated by the FDA as a breakthrough therapy for metastatic squamous and non-squamous non-small cell lung cancer (NSCLC) with PD-L1 expression which continued to progress on or after platinum-based chemotherapy or an FDA-approved EGFR or ALK targeted agent (Dang et al. 2016). The FDA approved pembrolizumab (Keytruda, Merck) as an adjuvant treatment for stage I.B (T2a ≥ 4 cm), II, or IIIA NSCLC after resection and platinum-based chemotherapy on January 26, 2023, based on KEYNOTE-091 (NCT02504372) Trial in which pembrolizumab demonstrated a significant improvement in disease-free survival in the overall population. Another mechanism by which mAbs can attack cancer cells is by attaching and blocking the antigens on cancer cells. For example, mAb trastuzumab binds to the HER2 receptor’s extracellular domain and inhibits HER2 homodimerization, inhibiting HER2-mediated signaling. It is also hypothesized to enhance antibody-dependent cellular cytotoxicity, resulting in the death of HER2-expressing cells (Greenblatt 2022). Trastuzumab was the first humanized mAb approved for HER2-positive metastatic breast cancer. It was approved by the FDA in 1998, which demonstrated to be an effective means of enhancing the prognosis and standard of care for chemotherapeutic regimens (Kreutzfeldt et al. 2020). It is also approved for metastatic HER2-positive breast cancer and HER2-positive gastric cancer (Greenblatt 2022). Margetuximab, a new monoclonal antibody derivative of trastuzumab, has an IgG1 Fc region genetically modified, which binds to the same epitope of the HER2 receptor as trastuzumab and has comparable antiproliferative effects. The advantage of margetuximab over trastuzumab is its affinity (6.6 times greater) towards stimulatory CD16A FcγRIIIIA on N.K. cells and about 8.4 times less for the inhibitory CD32B FcγRIIB found on immune effector cells (N.K. cells and macrophages) within the innate immune system which improves the host’s immunological identification of cancer cells beyond trastuzumab (Kreutzfeldt et al. 2020).

Moreover, monoclonal antibodies can also be attached to radioactive particles and chemotherapy medicines. Ibritumomab tiuxetan, for example, is a murine mAb directed against human CD20, and when coupled to a linker chelator it allows the inclusion of the short-lived β-emitter radioisotope yttrium 90 used against non-Hodgkins B cell lymphomas (Ross 2013). Ado-trastuzumab, an antibody-drug conjugate of trastuzumab and emtansine, is the first FDA-approved ADC for HER 2-positive breast cancer patients. The anti-tubulin capabilities of concentrated high doses of emtansine are combined with the anti-HER2 properties of Trastuzumab in ado-trastuzumab. The most significant benefit of ado-trastuzumab is the ability to administer chemotherapy to breast cancer tumor cells that are particularly HER2-positive. Direct delivery of cytotoxic drugs to cancer cells reduces toxicity on surrounding healthy tissue while also enabling the use of more effective chemotherapy (Isakoff and Baselga 2011). In phase III (EMILIA clinical trial), the Antibody-drug conjugate showed a higher objective response rate than lapatinib and capecitabine combination therapy (Verma et al. 2012). Based on the results of the EMILIA clinical trial FDA approved Ado trastuzumab in 2013 for patients with HER2-positive Metastatic breast cancer who received trastuzumab and a taxane separately or in combination previously (Wedam et al. 2020).

Similarly, bispecific antibodies are in development that can bind to multiple targets. For example, Blinatumomab, a bispecific antibody, comprises two linked single-chain variable antibody fragments. One of the fragments attaches to the TCR/CD3 complex and the other to the TCR/CD19 complex on B cells, generating an immunologic complex that causes the CD19-positive leukemia cell to undergo apoptosis (Elitzur et al. 2023).

### Limitations of Antibodies

After the advent of hybridoma technology in 1975, monoclonal antibodies became a potential category of therapeutics against malignancies, infectious diseases, autoimmune diseases, etc. However, certain limitations and drawbacks are associated with using antibodies. One of the major drawbacks of monoclonal therapy is an immune reaction (Presta 2006). Monoclonal antibodies from mice (mAbs) have proven incredibly helpful in diagnostics. However, short-lasting effects which are attributable to the development of human anti-mouse antibodies (HAMA) result in clearance of the murine mAb and might lead to fatal adverse effects (Hwang and Foote 2005). Similarly, technological advancements allow researchers to transition from potentially immunogenic murine monoclonal antibodies to chimeric, humanized, and fully humanized mAbs; still, immune reactions may occur through various mechanisms. Multiple mechanisms, including acute anaphylactic (IgE-mediated) and anaphylactoid responses to the mAb, serum sickness, tumor lysis syndrome (TLS), and cytokine release syndrome (CRS), might result in acute reactions after mAb infusion resulting in clinical symptoms. For instance, in a clinical trial (EXTREME trial) of cetuximab (a chimeric mouse-human IgG1 monoclonal antibody against EGFR), Cetuximab-induced infusion responses (CI-IR) occurred in 2.7% of the patients; however, many studies show higher rates (Palomar Coloma et al. 2018). Coloma et al., in their study, showed that 24 of the 428 patients (5.4%) presented CI-IR, including 95.7% Grade 3–4 anaphylaxis reaction, while 21% required intensive care referral (Palomar Coloma et al. 2018). In another study, Chung et al. tested whether severe hypersensitivity responses to cetuximab are caused by preexisting IgE antibodies against cetuximab. Interestingly, they found that among 76 patients treated with cetuximab, 25 developed hypersensitivity 13 (Grade 1 or 2) and 12 severe (Grade 3 or 4) to the drug, and among these patients, IgE antibody against cetuximab was found in 17 pretreatment samples. The oligosaccharide galactose-α-1,3-galactose on the Fab section of the cetuximab heavy chain was the target of these IgE antibodies (Chung et al. 2008).

Another drawback of monoclonal antibodies is their cost. According to Hernandez et al., the average yearly price of FDA-approved monoclonal antibodies from 1997 to 2016 was around $96,000. Similarly, the cost of mAbs in cancer was $98,000 greater than in immunology, $128,000 higher than in infectious diseases or allergies, and $106,000 higher than in ophthalmology (Hernandez et al. 2018). For a perspective, in time driven activity based costing (TDABC) analysis, which predicts costs by explicitly accounting for resources consumed and the time spent with each resource, the basic cost for two rituximab infusions was calculated around $19,000 in 2018 USD and drug cost accounted for more than 90% of the costs (Wallace et al. 2020). Similarly, Drucker et al. reported the cost per patient for adjuvant and palliative treatment of trastuzumab per patient in HER2 + breast cancer was $49,000 and $28,000, respectively, and the acquisition cost of mAbs was found to be more than 94% (Drucker et al. 2008).

To discuss further, the efficacy of monoclonal antibodies is also limited due to their physiological mechanism of action. In detail, monoclonal antibodies function due to the interaction of the Fc domain of antibodies with host Fcγ receptors. The members of Fcγ receptors are divided into three subgroups, including FcRI (CD64), FcRIIa, b,c (CD32a, b,c), and FcRIIIIa, b (CD16a, b), which are produced by immune effector cells such as macrophages, neutrophils, dendritic cells, and natural killer (NK) cells. The interaction between Fcγ receptors and the Fc region of the antibody leads to either phagocytosis of the target cell or release of perforin/granzyme causing apoptosis of the target cells (Chames et al. 2009). Studies have shown the association between the polymorphism of the Fcγ region and clinical responses to antibody therapy. In 20% of the white population, the FcγRIIIa has valine in position 158 (FcγRIIIa-V158) instead of phenylalanine (FcγRIIIa-F158). When it was tested, in vitro FcγRIIIa-V158 had a fivefold greater affinity for IgG1 Fc than FcγRIIIa-F158, resulting in more efficient Antibody-dependent cellular cytotoxicity (ADCC) employing peripheral blood mononuclear cells (PBMCs) or purified N.K.s (Chames et al. 2009). Furthermore, glycosylation is crucial in ADCC. The Fc region of IgG1 molecules is glycosylated at the CH2 domain (Asn 297) and has been found to alter FcRIIIa affinity. For example, the presence of fucose residues in the carbohydrate has been shown to decrease the ADCC efficiency (Shinkawa et al. 2003).

Furthermore, cytotoxicity and pharmacokinetics are issues when targeting cancer cells using antibody-drug conjugates and radioimmunotherapy. Although tumor-selective antibodies are more specific to tumor antigens, they can cause systemic toxicity, and many cancers are inaccessible to deliverable amounts of radiation. Additionally, neutralizing antibodies like immunotoxins may be generated against these antibodies, reducing their usefulness (Kreitman et al. 2009). Antibody proteolysis or fragmentation is another major problem. Yang et al. (Yang et al. 2019) discovered and categorized fragmentation of a therapeutic IgG4 mAb-X bin Chinese Hamster Ovary (CHO) host cell by residual protease impurities during the development of the early purification and formulation process. They solved this problem by optimizing the purification process to remove the protease, thus ensuring the stability and efficacy of the product. Furthermore, mAb aggregation is also another problem during processing. Arosio et al. found that pH and salt concentration are the main parameters that affect mAb aggregation. In the presence of salt, at pH less than 4, aggregation of the mAb is largely increased to form an oligomer, followed by an increase in the secondary β-sheet structure. However, when diluted in a salt-free solution, the monomer is recovered, which implies that the aggregation was reversible (Arosio et al. 2011).

### Peptide Therapeutics

There has been a constant revival of interest and specific momentum in peptide research since the discovery and development of the hormone insulin, representing the untapped potential of low-hanging fruit (Bliss 1984). The beginning of the 20th century was directed toward grasping the structures and physiological role of oxytocin, vasopressin, and gonadotropin hormone-releasing hormone (GnRH). It took nearly 60 years to introduce human insulin as a recombinant drug. Until the dawn of the 21st century, peptides as medicine were considered a niche area of drug discovery. However, since the 2000s, 28 new non-insulin peptide drugs have been approved worldwide, and more than 80 new peptide drugs from other research areas, mainly metabolic disease, and oncology (Lau and Dunn 2018), and more than 170 peptides, are in active clinical development (Henninot et al. 2018; Lau and Dunn 2018). These achievements caused the pharmaceutical industries to defy Eroom’s law, encouraging them to allocate more resources to explore and bolster the conceivable area of research (Henninot et al. 2018). Peptides act as hormones, growth factors, antibacterial agents, neurotransmitters, natural ligands for G-protein coupled receptors (GPCRs), ion channels, and growth factor receptors, exemplifying their critical role in human physiology (Fosgerau and Hoffmann 2015). Therefore, most peptide drugs are agonists of signal transduction pathways. These characteristics of natural peptides led to the generation of validated leads in drug discovery (Henninot et al. 2018). The therapeutic application of peptides is mainly restricted to extracellular and transmembrane targets as they are membrane impermeable, which excludes blood brain barrier (BBB) therapy; however, this field has progressed in oral (Mullard 2015; Aguirre et al. 2016; Maher et al. 2016; McCartney et al., 2016), and intracellular delivery: (Leeson and Springthorpe 2007; Arnott and Planey 2012; Nielsen et al. 2017). In addition, the short plasma half-life of peptides owing to proteolytic degradation and renal filtration has restricted its prominence in revenue generation. These shortcomings are avoided using various optimizable medicinal chemistry approaches such as D-amino acids, which prevent chymotrypsin degradation, and α-Me- amino acid, which impedes protease binding (Yamaguchi et al. 2003; Jo et al. 2012; Werner et al. 2016). N-Me- amino acid bids good protease protection and disrupts intra/intermolecular H-bonding, potentially decreasing unwanted aggregation, improving solubility, and increasing bioavailability (White et al. 2011; Chatterjee et al. 2013). Other strategies include cyclization, which restricts the conformational flexibility for optimum binding to the target(Di 2015); whereas in the case of helical peptides, cyclization of side chain-to-side chain has proven effective (Ahn et al. 2001; Hoang et al. 2015), bridging three cysteine residues (Chen et al. 2014a) and ring-closing metathesis produces hydrocarbon stapled peptides for designing cell-penetrating peptides (Bird et al. 2010). Many peptide conjugation avenues are used, such as side-chain modification (ornithine, homoarginine, citrulline, β-methyl group) (Haskell-Luevano et al. 1997; Wisniewski et al. 2011). The mentioned approaches gave rise to many peptidomimetics, which have emerged as alternatives to surmount the deficiency related to the intrinsic properties of peptides. Pelay-Gimeno et al. (2015) classified peptidomimetics into four groups based on the structural backbone modification of the peptides, i.e., Class A- minimal alteration to the natural amino acids, such as methylated proline or benzylated tyrosine (Cvoclosporin, pasireotide). Class B comprises unnatural amino acids (setmelanotide, bremelanotide). Class C represents amide bond replacement by triazole or oxetane rings or aza-linkages (benazepril, atanazavir), and Class D focuses on mimicking the mode of action of the natural peptides by small molecule drugs (Idasanutlin)(Pelay-Gimeno et al. 2015). Considering the mentioned attributes of peptides, it is imperative to note that the proteolytic instability and rapid clearance of peptides suggest that they donot accumulate in the tissue and hepatic metabolism is insignificant, which indicates that the drug-drug interaction or off-target effects are minimal, outsmarting the small molecule drugs. The other advantages include predicting human peptide doses, which are simpler, lower immunogenicity, and relatively simple drug discovery optimization (Kaspar and Reichert 2013). Another aspect to consider in synthesizing peptides is using genetic code expansion to produce noncanonical amino acid-containing proteins, pioneered by Hiroaki Suga (Chin 2014; Maini et al. 2016). The amalgamation of genetic code expansion and synthetic methodology will remove the peptide drug discovery constraint.

### Stable Peptides from Natural Resources

**Fig. 1:**
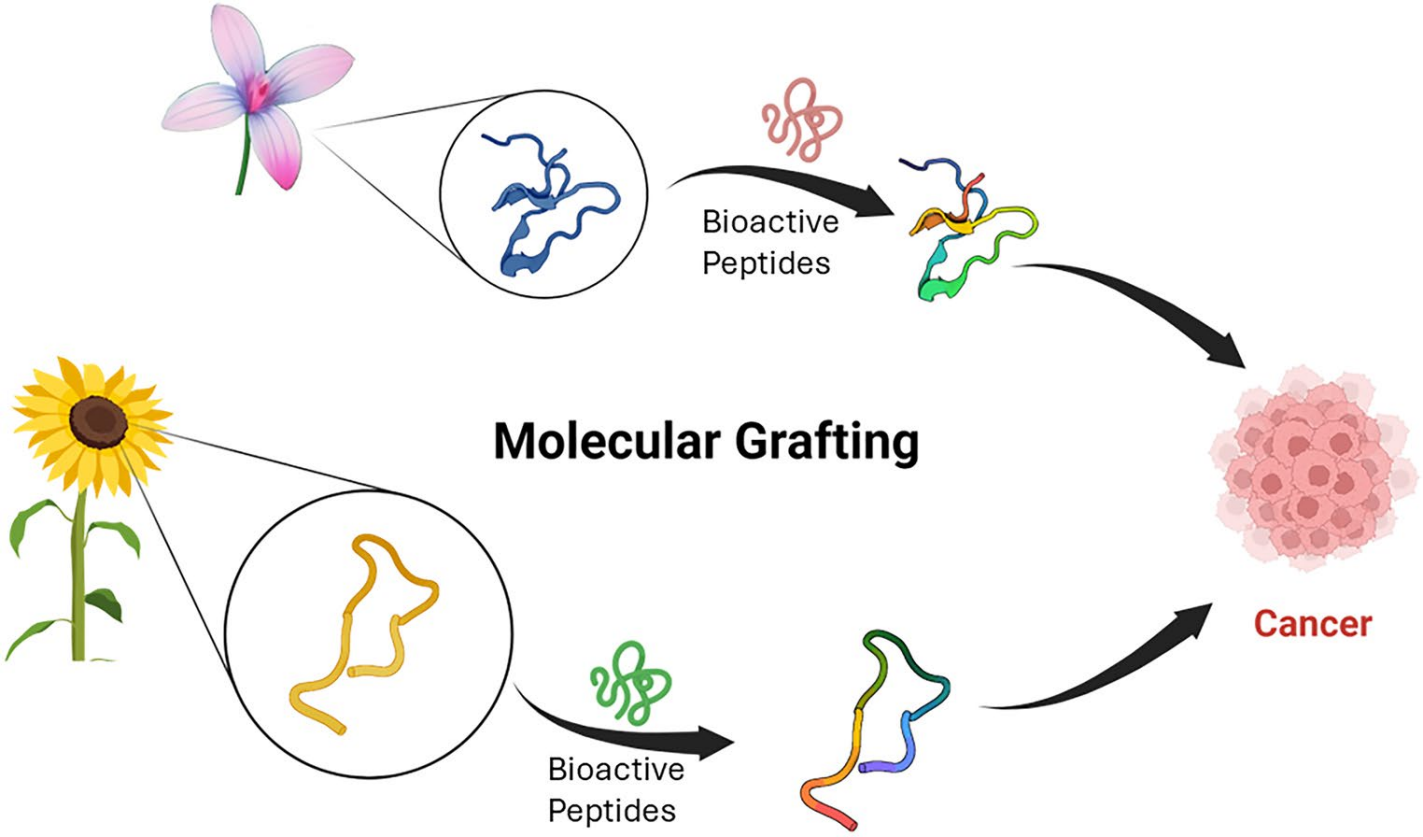
Bioactive stable peptides that can be engineered to have stability and pharmaceutical properties. Biorender was used to generate the figure

The plant kingdom has always fascinated researchers with its plethora of analeptic properties. These curative properties range from agricultural to drug leads or scaffolds for pharmaceutical drug design. Several bioactive peptides with anticancer effects are reported in the literature. A comprehensive list of bioactive peptides is provided in Ghadiri et al. (Ghadiri et al. 2024). This review investigates the class of plant-derived macrocyclic peptides called Cyclotides which are mostly 30 amino acid residues. They are typified by head-to-tail cyclic backbone and cystine knot motif, which makes them exceptionally resistant to thermal or enzymatic degradation (Fig. 1).

Plant species belonging to families such as Rubiaceae, Violaceae, Solanaceae, Cucurbitaceae, and Fabaceae flourished with cyclotides, uncommon in other plant species. Cyclotides are macrocyclic peptides of 28–37 amino acid residues (de Veer et al. 2019). The term “Cyclotide” was used for the first time in 1999 (Craik et al. 1999) which offered a unique structure defined by cyclic cystine knot (CCK) structural moiety (Saether et al. 1995). The CCK motif embraces a head-to-tail cyclic backbone with a cystine knot formed by six conserved cysteine residues. The first cyclotide to gain prominence was Kalata B1 from *O.affinis*, used in Congo for its uterotonic activity. Due to the absence of any chemical or genetic means in the 1970s, the structure and composition of Kalata B1 were unknown (Gran 1973). However, in 1995 the CCK moiety was delineated using either bioassay-guided or mass-guided fractionation of plant extracts, peptide isolation and purification, Edman degradation, NMR (Saether et al. 1995), and mass spectrometry (Derua et al. 1996) based strategy to extrapolate the disulfide connectivity. The initial goal to identify whether a plant contains cyclotide was based on the extraction process using dichloromethane (DCM)/methanol, aqueous acetonitrile (ACN), followed by the C18 reversed-phase, where a 348 Da mass shift after reduction and alkylation proved six cysteines residues (Gruber et al. 2008). Nearly 500 natural cyclotides with varying amino acid sequences have been isolated and a database is available, particularly for cyclotides (http://www.cybase.org.au) (Wang et al., 2008). Many of these natural cyclotides have shown potency in inhibiting cancer cell growth (Mehta et al. 2020).

### Structure of Cyclotides

As mentioned, cyclotides are categorized into three subgroups: Mobius, Bracelet, and trypsin inhibitor, with two-thirds being bracelet type, one-third being Mobius type, and a relatively small number of trypsin inhibitors. Mobius and Bracelet have a hydrophobic patch on the surface of their structure, which is attributed to the membrane binding and late elution in HPLC. The above-mentioned hydrophobic patch is placed close to conserved Glu residue in loop 1 (Shenkarev et al. 2006, 2008; Wang et al. 2009a, b) (as shown by NMR. common trait sequence in cyclotides can be represented by [C1-Xa-C2-Xb-C3-Xc-C4-Xd-C5-Xe-C6-Xf] with Xa, Xb, Xc, Xd, Xe, Xf being 3–6/4–5/3–7/1/4–6/5–8 residues respectively (Mulvenna et al. 2006; Wang et al. 2008; Kaas and Craik 2010). The amino acid content in the cyclotides is entirely made up of proteinogenic amino acids, with cysteine being the most abundant and least abundant across all-natural peptide and protein sequences. Next, the plentiful amino acid is proline, even higher than most proteins, while alanine, histidine & methionine are marginal. Apart from head-to-tail cyclization and disulfide bonds, cyclotides do not contain any other post-translational modification. However, recently hexose sugar was found to be attached to lysine residue in cyclotide from the Violaceae family (Burman et al. 2015). In *Melicytus macropyllus* and *M. ramiform*, hexose moieties were associated with loop 5, which was proved by MS/MS and NMR spectroscopy. In addition, recent reports suggest that the range of PTMs in cyclotides from *C.ternatea* included glycosylation, dehydration, deamidation, oxidation, hydroxylation (Serra et al. 2016). Details of the structure and functional aspects of cyclotides are described in several reviews and work from Dr. David Craik’s group. Some of the cyclic peptides (Costa et al. 2023), including cyclotide-based peptides (Gründemann et al. 2019) that are in therapeutic use, are listed in Table 1. Table 2 lists some cyclic peptides and conjugates that are in clinical trials for cancer (Costa et al. 2023).

**Table 1:** Stable cyclic peptides and cyclotide scaffold used targeting different proteins for therapeutic purposes

Name	Mechanism	Cyclization type	Therapeutic application	reference
Ziconotide	A blocker of N-type calcium channels	Three disulfide bonds	Severe chronic pain	(Nielsen et al. 1996; Lin et al. 2024)
Linaclotide	Guanylate cyclase 2 C agonist	three disulfide bonds	Irritable Bowel Syndrome with Constipation (IBS-C)	(Taclob et al. 2024) (Hornby 2015)
Plecanatide	Guanylate cyclase C agonist	Two disulfide bond	IBS-C & Chronic Idiopathic Constipation	(Brancale et al. 2017; Bassotti et al. 2019)
Pasireotide	Blocks GH, TSH, insulin, Glucagon	Head-to-tail cyclization	Cushing’s Syndrome acromegaly	(Puig-Domingo et al. 2021; Bolanowski et al. 2022)
Setmelanotide	Selective Melanocortin-4-receptor (MC4R) agonist	Disulfide linkage	Severe obesity caused by genetic disorders including deficiency of POMC, and PCSK1	(Markham 2021; Hammad et al. 2022; Pressley et al. 2022)

**Table 2:** Some cyclic peptides under clinical investigation for various types of cancer

Name	Mechanism	Cyclization type	Indication/Clinical trial	reference
POL6326	Inhibits chemokine receptor CXCR4	Backbone and disulfide bond cyclization	Advanced breast cancer/Phase I (NCT01837095)	(Karpova et al. 2017)
BL-8040	high-affinity antagonist for CXCR4	Disulfide bond cyclization	HSCs, solid tumors and AML Phase IIa with Pembrolizumab, nelarabine, motixafertide (NCT02826486)	(Rebolledo-Bustillo et al. 2023)
BT1718	targets membrane type-1 matrix metalloproteinase (MT1-MMP)	Cysteine cyclization via organic linker Conjugated to toxin	MTI-MMP expressed cancer Phase I/II (NCT03486730)	(Cook et al. 2019; Mudd et al. 2022)
BT8009	targets Nectin-4	Cysteine cyclization via organic linker Conjugated to toxin	Nectin-4 expressed cancer Phase I/II (NCT04561362)	(Mudd et al. 2022)
BT5528	Targets tumor expressed antigen EphA2	Cysteine cyclization via organic linker Conjugated to toxin	Advanced solid tumors Phase I (NCT04180371)	(Bennett et al. 2020; Mudd et al. 2022)
VT1021	Stimulates the expression of thrombospondin-1 (TSP-1)	backbone	Advanced solid tumors Phase I (NCT03364400)	(Mahalingam et al. 2024)

### Functional Application

**Fig. 2:**
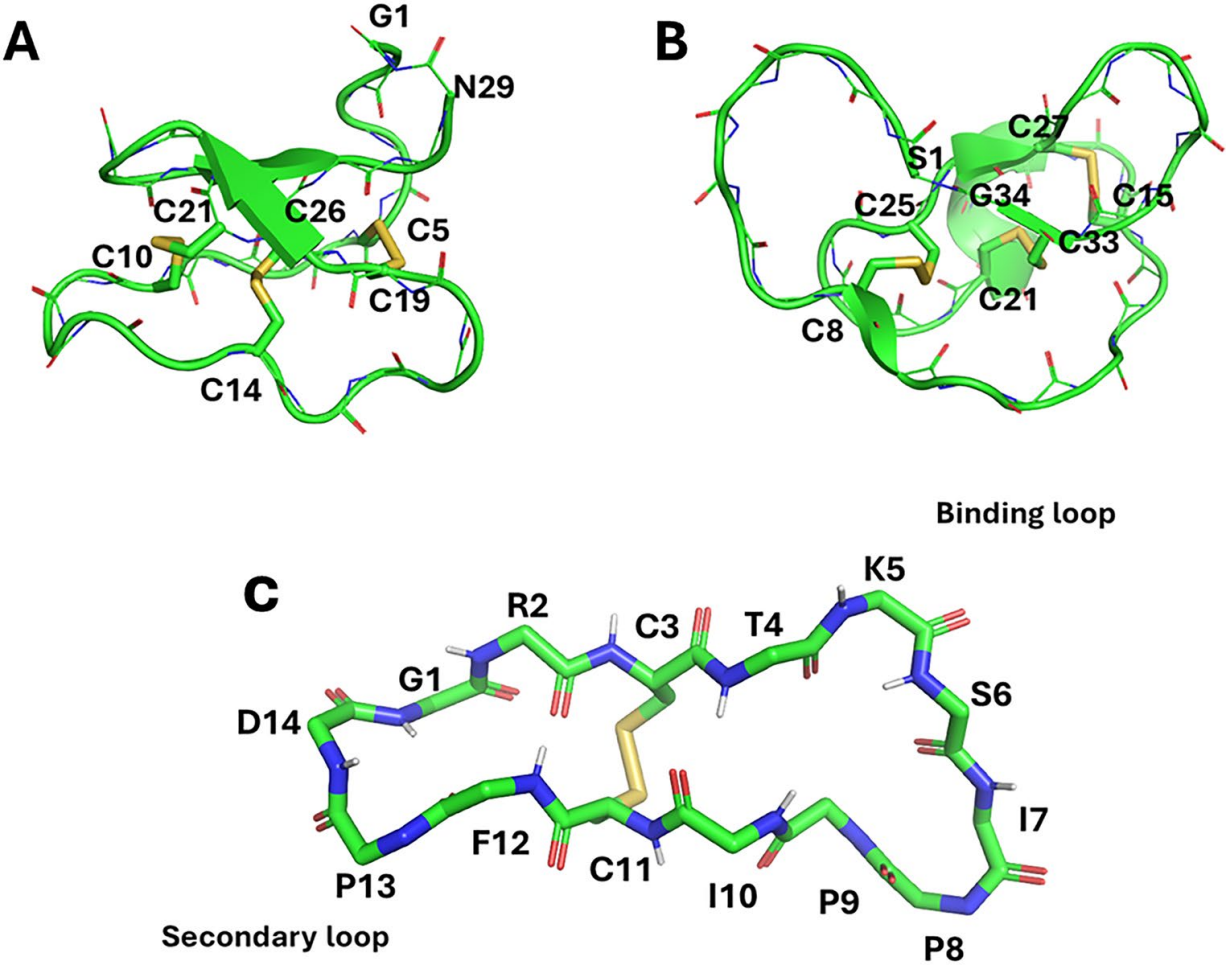
Structures of kalata B1 (PDBID: 4TTN)(Wang et al. 2014), MCo-TI-II (PDBID: 4GUX) (Daly et al. 2013) and SFTI-1 (PDBID: 1JBL)(Korsinczky et al. 2001) indicating disulfide bonds and loops

The distinctive chemical structures and functions of various classes of cyclotides have paved a new era to harness their property to develop novel biopharmaceuticals and insecticides for agricultural purposes. In addition, the bioactivity of cyclotide has been used to create new drug leads, which Kalata B1 can exemplify (Fig. 2A). Kalata B1 inhibits T-cell proliferation, which prompted its use as an immunosuppressive agent (Grundemann et al. 2012). However, the various mutants of Kalata B1, such as G18K, T20K, and N29K, retained antiproliferative activity (Gründemann et al. 2013). Hellinger et al. showed that inhibition of T-cell proliferation diminished IL-2 signaling and lowered secretion of IFNγ and TNFα (Hellinger et al. 2017), which was proved using a photoreactive crosslinking probe bound to Kalata B1 in T-cell lysates, altering Akt/PI3K/Foxo3a signaling. Among the variants of Kalata B1, the immunosuppressive activity of T20K was assessed using the EAE model, where the inhibition of T-cell proliferation was shown ex-vivo along with IFNγ and IL-17 A (Thell et al. 2016). Histological analysis showed that T20K inhibited the infiltration of immune cells into the spinal cord and prevented demyelination, making T20K its way to be evaluated for phase I clinical trial by Cyxone (Gründemann et al. 2019). As mentioned before, cyclotide concentration varies in different parts of the plant. For example, in butterfly peas (Nguyen et al. 2011; Poth et al. 2011), seeds have more cyclotides than other parts. These findings suggest that cyclotides are expressed differently in different tissues where they protect the plant from pests and nematodes(Gilding et al. 2016), resulting in the commercialization of the bioinsecticide Sero-X in Australia.

### Redirected Functions

The redirected function of the cyclotides has been prominently found in the MCoT-II. These trypsin inhibitors target serine proteases, and many natural genetic manipulations are used for other purposes. Like MCoTI-II (Fig. 2B), which is specialized to bind within the active site of trypsin, it represents a valuable scaffold for targeting structurally related proteases. In addition, MCoTI-II loops are highly complaint for sequence modification in many loops (loops 1,2,3). As proteases are intracellular enzymes, the easy accessibility of MCoTI-II intracellularly makes it an asset for specific protease inhibitors. Substituting key residues results in improved activity against new proteases. Sommerhoff et al.(Sommerhoff et al. 2010) and Avrutina et al (Avrutina et al. 2008) showed that a tripeptide Lys-Lys-Val was substituted in loop 6 of MCoTI-II and a hybrid MCoTI-knottin generated β-tryptase inhibitors in the nanomolar range respectively. However, engineering loop six by deleting four residues (Ser-Asp-Gly-Gly) retained β-tryptase activity while abrogating trypsin inhibition (Thongyoo et al. 2009). The enzyme matriptase, a membrane-anchored protease that is overexpressed in cancer, is inhibited by engineered MCoTI-based inhibitor by substituting P4 Val for Arg, P1’ Ile to Ala replacement resulted in higher affinity for matriptase and decreased the inhibitors off-target activity against trypsin (Quimbar et al. 2013). MCoTI-II’s substrate-like mechanism was utilized to develop an inhibitor for coagulation factor XIIa (Swedberg et al. 2016). To further support the redirected functionality of MCoTI, Swedberg et al (Swedberg et al. 2018). demonstrated a substrate-guided approach for kallikrein-related peptidase 4, a protease that is overexpressed in prostate cancer. When P1, P2, and P4 were substituted, exceptional selectivity was observed for closely related proteases.

Another milestone application of cyclotides was achieved when a chimeric peptide was designed by a technique called grafting. This method allows the production of a chimeric peptide with an ultra-stable cyclotide scaffold and an epitope of interest. As cyclotides are exceptionally tolerant to sequence modification, it becomes an easier target for chemical engineering to produce a range of new functionalities. In addition, grafted peptides have the unique property of being efficiently orally active, which offered a clue in Kalata B1 when consumed after brewing, suggesting a uterotonic effect (Wong et al. 2012) this oral activity of cyclotides can be typified by the study involving bradykinin B1 receptor antagonist as a treatment for chronic pain which was produced by efficiently grafting nine amino acids epitopes to loop six of Kalata B1 (Wong et al. 2012). The analgesic activity of this variant was proved using an animal model where the grafted cyclotide offered more pain relief than the linear B1 receptor antagonist. However, there needs to be more oral bioavailability and pharmacokinetic studies for grafted cyclotides. Cyclotides have been utilized to deliver cargoes inside cells, i.e., MCoTI-II, which are easily internalized in the cells. This approach modulated the p53 pathway by blocking the binding of Mdm2/x (Ji et al. 2013). Trypsin inhibitors were chosen for this study as they quickly internalize and don’t negatively affect membranes. Here, the chimeric peptide was designed by grafting α-helical peptide that binds Mdm2 and MdmX into loop 6 of MCoTI, showing activity at nanomolar concentration. Grafted peptides were even used to enhance their selectivity property, like selective targeting of a range of GPCRs. Here, loop 3 of Kalata B7 closely resembles human oxytocin (Koehbach et al. 2013), activating the oxytocin receptor.

Further experiments showed that residues 14–22 of Kalata B7 in loop 3 displayed higher activity. Aboye et al. showed that a 14 amino acid epitope was grafted to MCoTI-I in loop 6 to produce an antagonist of chemokine receptor CXCR4, blocking the entry and replication of HIV-I in MT4 lymphocytes (Aboye et al. 2012). When conjugated to 1,4,7,10-Tetraazacyclododecane-1,4,7,10-tetraacetic acid (DOTA), this grafted peptide was used as a PET-CT imaging agent (Lesniak et al. 2017).

Cyclotides’ size, topology, and molecular weight have become an entity with many promising applications in biopharmaceuticals and agriculture. It covers an area of molecular weight between small molecules and biologics, with exceptional stability and amenability for scaffold engineering. Furthermore, as these are plant-derived peptides, manufacturing can be done by using plants as biomanufacturers for producing pharmaceuticals and agrichemicals like the Kalata B1 variant, T20k produced in transgenic plants (Poon et al. 2018), and a picomolar plasmin inhibitor based on SFTI-1 (Jackson et al. 2019). In addition to providing a greener way to produce biopharmaceuticals and agrochemicals, it also offers a potential novel delivery route in terms of biopills, giving potential for the economic supply of next-generation medicines, overcoming the limitations of cold-chain transport of conventional peptides and protein formulations. Moreover, a recent approach for cyclotides as nanoparticles that exhibited antitumor activity and sustained drug release is gaining importance (Silva et al. 2019).

### Sunflower Trypsin Inhibitor-1 (SFTI-1)

The vastness of medicinal properties provided by cyclic peptides furnishes inspiration for the future engineering of chimeric peptides using drug discovery and synthetic chemistry. For example, sunflower trypsin Inhibitor-1, one of nature’s smallest known disulfide bridged cyclotide, has an atypical biosynthetic pathway that incepts with a dual-purpose albumin precursor and terminates with a high-affinity serine protease inhibitor. Nature is inundated with cyclic peptides like θ-defensins, cyclic bacteriocins, and cyclomarins (Schultz et al. 2008; Montalbán-López et al. 2012; Conibear and Craik 2014). Moreover, the cyclic backbone provides an evolutionary advantage of being refractory to the digestive medium. Most commonly, the kingdom Plantae is a rich source of cyclotides, mainly in Rubiaceae, Violaceae, Cucurbitaceae, Fabaceae, and Solanaceae (de Veer et al. 2019), consisting of three disulfide bonds with 28–37 amino acids residues, synthesized in the ribosome (Craik et al. 1999). The discovery of Kalata B1, the cyclotide from O.affinis, paved the way for the emergence of the cyclotide field in 1999 (Craik et al. 1999). However, SFTI-1 defied the definition of cyclotide by being a 14 amino acid residue showing potent inhibition against serine protease trypsin (Luckett et al. 1999). Once the SFTI was identified, it was imperative to elucidate its strenuous structure as it had head-to-tail cyclization, making it impossible to be determined by Edman degradation. However, the X-ray crystallography of SFTI-1 with β-trypsin bound revealed that it consisted of two antiparallel β strands connected by type VIb β at one end and a type 1 β turn at the other (Luckett et al. 1999). The SFTI-1 consists of two loops; the primary loop (Thr4-Ile10) is the largest and responsible for protease inhibition (Fig. 2C). The other is the “cyclization loop” (Phe12-Arg2) that contains the biosynthetic cyclization amino acids (Gly1-Asp14) residues, along with a disulfide bond between Cys3 & Cys11, two prolines at position 8 and 9 in *cis* and *trans* configuration respectively. In addition, an immense network of intramolecular H-bonds between amino acids’ main and side chains. Korsinczky et al. in 2001 (Korsinczky et al. 2001) showed several NOE connectivities indicating an antiparallel β-sheet with Thr4, and Phe12 contributed in cross-strand H-bonds and Gly1, Arg2, Ile10 were involved in loop stabilizing H-bonds. The SFTI-I’s free and trypsin-bound structure revealed the inhibitor’s binding specificity, i.e., Arg2-Ile7 is the primary critical contact with the scissile bond between Lys5, and Ser6 (Schechter and Berger 1967). SFTI-1 has a distinctive, well-defined conformation similar to the Laskowski inhibitor (Laskowski and Kato 1980). Mechanistically, these inhibitors offer a fascinating approach whereby the protease cleaves the inhibitor at the scissile bond to generate an acyl-enzyme complex where the binding loop adopts a conformation of a favored substrate. Once the new N-terminus results at P1’s position, which is held in place by intramolecular interaction within the inhibitor core preventing hydrolysis of the acyl-enzyme bond. Instead, the neo-N-terminus is activated by the catalytic histidine residue instead of the water molecule, re-ligating the scissile bond and regenerating the inhibitor, which Marx et al. proved in 2010 (Marx et al. 2003; Colgrave et al. 2010).

SFTI-1 is a part of the 151-residue dual-purpose precursor protein, which encodes a 2 S-type seed storage albumin protein called PawS1(Mylne et al. 2011). The small and compact size of SFTI-I was used for the peptide design/engineering using different chemical syntheses approach. As it has a single disulfide linkage, it becomes easier to incorporate non-canonical entities to design peptide scaffolds as needed, resulting in the designing protease inhibitors by molecular Grafting. The atypical tolerance of the SFTI-1 framework to sequence modification resulted in the creation of peptides for different protein targets, mainly operable in inhibiting protein-protein interaction.

### SFTI-1 as a Molecular Grafting Scaffold

The sequence modification tolerability of SFTI-I has been a magnificent attribute leveraged by chemists worldwide to construct molecular entities with significant activities towards different targets, enhancing the chemical space for drug discovery. The molecular grafting framework involves incorporating a peptide epitope with desired bioactivity into an ultra-stable peptide scaffold, generating a chimeric molecule that possesses the grafted epitope’s biological function and the platform’s inherent stability, increasing overall drug-like properties (Wang and Craik 2018). Jaulent et al. (Jaulent and Leatherbarrow 2004; Jaulent et al. 2005) showed when the cyclization loop was replaced with a second repeat of the primary coil of SFTI-1, it produced a bifunctional protease inhibitor maintaining a rigid conformation in solution with no cis-trans isomerization at Pro residues, demonstrating the tolerability of SFTI-1 to sequence alterations. Many studies have shown that SFTI-I scaffold can bear sequence alterations in the primary loop, cyclization loop, and the β-strand, with the primary coil being altered in most cases (Chan et al. 2011; Zoller et al. 2012; Gunasekera et al. 2018; Roesch et al. 2018). Diverse epitopes can be grafted into SFTI-1 without impairing the scaffold’s metabolic stability, which creates a drug design perspective. For instance, SFTI-1 grafted peptide analogs were developed to promote angiogenesis by targeting integrins or VEGF (Chan et al. 2011). Epitopes derived from laminin and osteopontin were grafted into the primary loop of SFTI-1, showing higher stability in human serum over 24 h. In contrast, epitopes derived from thrombospondin-1, somatostatin, or pigment epithelium-derived factors showed potent anti-angiogenic activity.(Chan et al. 2015, 2016) SFTI-1 was used in radiolabeling study to target αv β6 integrin for developing radiotherapeutics (Altmann et al. 2017; Roesch et al. 2018). An octapeptide epitope derived from latency-associated peptide three was grafted into the primary loop of SFTI-1, and the N-terminal was attached to the DOTA chelator. It was shown using PET that the peptide accumulated in primary and metastatic tumors (Roesch et al. 2018). Protein-protein interaction inhibition is a most sought-after target for drug discovery, which uses SFTI-1 scaffold for many disorders like cancer, rheumatoid arthritis, Alzheimer’s, and cardiovascular diseases The versatility and sequence modification accessibility, yet retaining structural and metabolic activity, have proven to be an essential avenue in drug discovery, indicating a future inundated with diagnostics and therapeutics.

Integrins are cell adhesion receptors and these cell surface protein receptors play an important role in the development of cancer. Among integrins αvβ6 integrin is known to be upregulated in different types of cancers. A small peptide stretch of three amino acids, namely, Arg-Gly-Asp (RGD) (Pierschbacher and Ruoslahti 1984) is important in binding integrin to extracellular matrix proteins such as fibronectin, vitronectin, and latency-associated peptides (LAP)1 and 3. The RGD-containing peptide was grafted to SFTI-1 framework to increase the stability of the RGD peptide. The peptide SFLAP3, exhibited binding to αvβ6 integrin that is expressed in head and neck squamous cell carcinoma (HNSCC) with high affinity. This peptide was labeled with 177Lu using DOTA. Using cellular uptake studies and PET scans in the clinical setting, it was shown that SFTI-1 grafted peptide can be used as an imaging agent in cancer diagnosis (Roesch et al. 2018). In our laboratory, we have used sunflower trypsin inhibitors for grafting a peptide that exhibited antiproliferative activity in HER2-positive cancer cell lines. With the grafting approach, we were able to design a peptide that is stable in vivo and was able to suppress non-small cell lung cancer in xenograft models of cancer in mice (Singh et al. 2021).

### Summary and Future Directions

Biologicals such as antibodies and fusion proteins are highly specific for their target proteins. For the past three decades antibody therapy has revolutionized targeted therapy and there are more than hundred monoclonal antibodies approved by the FDA for the treatment of different human diseases. The major area of antibody therapy is for cancer and autoimmune diseases including cancer immunotherapy. Antibody therapy regained momentum when antibody drug-conjugates such as Ado-Trastuzumab emtansine, and brentuximab vedotin was approved for therapeutic use (Jin et al. 2022). One of the major limitations of antibody drugs is the immunogenicity and delivery methods and stability at room temperature. In particular for cancer therapy antibodies develop resistance. In cancer tumors, genetic alterations that happen due to microenvironment or treatment with drugs lead to the development of acquired resistance modify the cellular phenotype, and undermine the initial therapeutic efficacy (Pallasch et al. 2014; Baker et al. 2018). The tumor microenvironment poses physical barriers, limiting the penetration of macromolecules into the tumor following systemic administration (Cruz and Kayser 2019). Thus, the local concentration of antibodies can be a suboptimal therapeutic dose, leading to acquired resistance and treatment failure (Thurber et al. 2008). In solid tumors, the lymphatic drainage system is irregular, accumulating large proteins such as antibodies in the interstitial tissue, creating hydrostatic pressure. The development of resistance is not fully understood. The cost of production of antibodies is also comparatively high, and most of the production is done in mammalian cells and hence susceptible to infection of bioreactors. Recently there has been production of some proteins using plant cells for therapeutic purposes. However, the number of plant cell-based protein therapeutics is limited. Thus, for targeted therapy, new and novel molecules are needed.

Peptide therapeutics (see FDA guidelines for details) are molecules with less than 40 amino acids (Craik and Kan 2021) falls between the two major categories of therapeutics—biologics and small molecules (5000 Da)(Wang et al. 2022). Peptides exhibit the high specificity and efficacy of biologics since many peptides are natural endogenous ligands for protein receptors. The production cost of peptides is relatively low compared to antibody therapeutics (Fosgerau and Hoffmann 2015; Tyler et al. 2023; Jois 2022). However, linear peptide sequences are digested by enzymes in the gut and at the brush border membrane and have limited stability in the low pH of the stomach acid when given orally. Upon intravenous (IV) administration they can be degraded by enzymes in the circulation and near the cells. One novel approach to overcome this shortcoming is to use the stable molecular scaffolds of peptides from nature and replace some of the amino acids in the stable peptide molecules with bioactive linear or cyclic peptides, generating grafted products with the desired properties of both parent compounds. The chimeric approach used in grafting can be used to include drug-like properties into the stable peptide framework, such as cyclotide and SFTI-1 framework. Due to their exceptional stability and desirable drug-like properties, cyclic disulfide-bonded peptides are attractive frameworks for designing peptides for therapeutic purposes (Zhang et al. 2021). Cyclotide and sunflower trypsin peptide scaffold can be used to graft the peptide. Cyclotides can cross cellular membranes, allowing them to target intracellular targets (Contreras et al. 2011; Jacob et al. 2022). These peptides are also highly resistant to the harsh acidic environment of the gastrointestinal (GI) tract and to the presence of proteolytic enzymes (Clemente et al. 2008; Cruz-Huerta et al. 2015).

The disulfide bonds present in cyclotides and sunflower trypsin inhibitors make them thermally and enzymatically stable and can tolerate a wide pH range (Gitlin-Domagalska et al. 2020)^,^(Kumar and Gowda 2013). The stable nature of cyclotides depends on the source and type of folding. For example, Bowman-Birk inhibitor (BBI) has been reported to retain 75% of its activity after 360 min incubation at 100 ^º^C. On the other hand, the soy extract peptide loses its ability to inhibit trypsin and chymotrypsin faster under similar conditions (Chen et al. 2014b; Gitlin-Domagalska et al. 2020). Since cyclotides are much smaller than proteins, with the progress in solid-phase peptide synthesis, they are suitable for chemical synthesis. Chemical modifications such as non-natural amino acids can easily be introduced in these molecular scaffolds to improve their pharmacological properties. Cyclotides can be expressed in several expression systems, and biotechnology can be used to generate peptides of various sequences for the selection of compounds with optimal binding and inhibitory characteristics (Gould and Camarero 2017). These novel characteristics make them promising candidates for peptide drug design. Even some cyclotides have been shown to be orally active. It is anticipated that more studies on the biopharmaceutical properties of these interesting micro-proteins will be available in the coming years.

There are reports of modifying the SFTI-1 framework for grafting several templates. Asymmetrical bifunctional protease was designed by modifying the SFTI-1 framework, and the peptide maintained a rigid conformation in solution without any cis-trans isomerization as observed in many SFTI-1 grafted peptides (Jaulent and Leatherbarrow 2004; Jaulent et al. 2005). This study demonstrated that the loop region of SFTI-1 could be replaced without any change in the SFTI-1 structure (de Veer et al. 2021). Our group and others have shown that proline amino acids in SFTI-1 framework can be replaced with organic functional groups while still retaining the biological activity (Tischler et al. 2012; Singh et al. 2021; Dahal et al. 2022). Examples of SFTI-1 framework that were tested in a human clinical setting, such as DOTA attached with SFTI-1 grafted peptide, provide evidence that molecular chimera using a stable framework can be used clinically, and this opens up new direction beyond antibody conjugates and imaging agents (Chan et al. 2016; Gunasekera et al. 2018; Roesch et al. 2018).

The chemistry of synthesis and cyclization is well established in the production of stable grafted peptides. Most of the synthesis can be carried out in an automatic peptide synthesizer, and disulfide bond formation needs to be carried out in a solution phase. Another exciting development in this regard is the production of SFTI-1 using a plant expression system. Poon et al. and Jackson et al. (Poon et al. 2018; Jackson et al. 2019) have shown that an engineered plasmin inhibitor peptide could be produced in a plant expression system, and 60 µg per gram of dry leaf weight can be produced. Such efforts can produce stable peptides in large amounts with low cost and green chemistry. The only limitation will be in the case of multiple disulfide bonds; there are different possibilities of disulfide bond formation, resulting in a low yield of the final desired peptide. However, with new developments in molecular biology techniques and synthetic approaches, the synthesis and production of cyclotides with multiple disulfide bonds will be optimized.

## Data Availability

No datasets were generated or analysed during the current study.
